# Stereotypes about tourette syndrome and associations with social media use: results from a large-scale german population survey

**DOI:** 10.1038/s41598-026-66366-8

**Published:** 2026-08-28

**Authors:** C. Cramer, S. Schmitt, N. Szejko, K. R. Müller-Vahl

**Affiliations:** 1 Clinic of Psychiatry, Social Psychiatry and Psychotherapy, Hannover Medical School, Hannover, Germany; 2 Department of Psychiatry, Psychotherapy and Psychosomatics, RWTH Aachen University, Aachen, Germany; 3 Institute for Translational Neuroscience and Clinical Psychology, RWTH Aachen, Aachen, Germany; 4 Department of Health Sociology, Education and Medical Communication, Institute of Mother and Child, Warsaw, Poland

**Keywords:** Social media, Tourette syndrome, Functional movement disorder, Stereotypes, Functional tics, Media misrepresentation, Diseases, Health care, Psychology, Psychology

## Abstract

Misunderstandings about tic disorders are common in both the media and the genreal public. However, no large-scale population-based study has investigated public knowledge and stereotypes about tic disorders. We conducted such a survey about knowledge and stereotypes about Gilles de la Tourette syndrome (GTS) in Germany in 2024. Among respondents who had heard about GTS (*n* = 1,175, 16–88 years, *M* = 47.9 ± 16.3), 50% of knowledge items were answered correctly. A score summarizing stereotyping statements revealed an average agreement of 39%. The vast majority associated GTS with coprolalia (87%), copropraxia (75%) and – less frequently – with other symptoms that are atypical but frequently portrayed in the media, such as contextualized commentaries (50%) and destroying objects (29%). Subjects who knew someone with GTS had better knowledge than those who did not know anyone with GTS. Regression analyses revealed a significant association between the estimation of own knowledge on GTS as good and stereotypical beliefs. Social media use to get informed about psychiatric diseases was linked to more knowledge and less stereotypes, however, having watched a video depicting individuals with presumed GTS was associated with more stereotypes. Our study demonstrated overall poor knowledge and widespread stereotypes about GTS in Germans. Studies evaluating options for stigma reduction are needed to foster equality and prevent disadvantages for affected persons.

**Supplementary Information:**

The online version contains supplementary material available at https://doi.org/10.1038/s41598-026-66366-8.

## Introduction

Primary tic disorders, including Gilles de la Tourette syndrome (GTS), are neuropsychiatric disorders characterized by motor and/or vocal tics with onset in early childhood^1^. Tics are involuntary, sudden, rapid, recurrent, non-rhythmic movements and/or vocalizations that are performed without purpose^2^.

There are several misunderstandings about various aspects of tic disorders, not only among the general public^3^, but also in persons with tics, their caregivers and - even more concerning - among members of advocacy groups and clinicians^4^. Unsurprisingly, in (social) media there are also severe misconceptions about tic disorders^5^. These false and potentially stereotyping beliefs have already been broadly described by other researchers. For instance, an online consumer panel survey conducted among 673 South Korean residents revealed misconceptions – such as the belief that GTS is caused by dietary habits, parenting practices, or insufficient discipline^6^. Another study examined the beliefs of parents whose children suffer from GTS. The results showed that many parents felt guilty and responsible for their children’s condition and, in some cases, attributed the cause of the tics to their children’s bad behavior or lack of mental stability^7^. A study investigated media representations of GTS in 37 international movies and television shows dated from 1976 to 2010 and found an overrepresentation of coprolalia, which in fact is a rare form of a complex vocal tic only present in about 8% of adult patients with GTS and in about 5% of youth with primary tic disorders^9^. In another study^10^, YouTube videos released until 2010 were analyzed that were labelled as showing people with GTS. The authors found that 22% of the 41 most watched videos depicted GTS negatively. Again - and contrary to current knowledge - coprolalia was often highlighted as the hallmark of the disease and half of the videos were amateurish and factually incorrect with mostly parodic or satirizing content^8,10^. Remarkably, negative portrayals were associated with even more views and comments in comparison to neutrally or positively connoted videos. Other authors^11^ analyzed 100 most watched TikTok videos allegedly showing people with GTS released before 2022. They also reported that the vast majority of videos focused on coprophenomena as well as other behaviors atypical for or rare in GTS such as aggressive and self-injurious behaviors or sentences with more than three words. A similar study analyzed videos of the most popular TikTok influencers having > 100,000 followers (with a total of 35.9 million subscribers and more than 331 million videos views) during the COVID-19 pandemic. Again, most videos were showing severe and atypical symptoms such as movements and vocalizations with long symptom duration lasting minutes to hours and high complexity often labelled as “tic attacks” making patients incapable of acting^12^.

Today it is widely accepted that in many videos shared on social media, people with functional tic-like behaviors (FTLB) rather than tics are portrayed^5,10,12,13^. It can be assumed that both increasing interest in GTS during the COVID-19 pandemic and widespread inaccurate digital portrayals of GTS significantly influenced public opinions about GTS causing even more stereotypes and as a result more stigma^14^.

When investigating stigma, sociologists have highlighted the importance of considering power relations between affected individuals and those in positions of authority including clinicians, teachers, and advocacy groups in influencing, for example, treatment and well-being^15^. In this context, a study from the Midwest of the US investigated beliefs of physicians and psychologists about GTS. However, only 67 of 443 distributed questionnaires (15%) were returned suggesting that results may be biased since only professionals with substantial knowledge on GTS could have responded. While participants answered 77% of general knowledge items on diagnosis, prevalence, etiology, course, and treatment correctly^4^, misbeliefs were common including beliefs that allergies or diets may affect tics, that tics can be suppressed completely, and that tic suppression results in a rebound of tics. In another survey from Saudi Arabia including 375 medical students and physicians, 66% were aware of the correct definition of tics, but knowledge of established treatment strategies for tics varied, with, with 46% recognizing antipsychotic medication and 74% habit reversal training as treatment options^16^. In a small study from the United Kingdom, teachers’ knowledge of GTS was investigated using semi-structured interviews (*N* = 8)^17^. The interviews revealed substantial lack of knowledge, since teachers described vocalizations and loud screams including the expression of profanities as most typical symptoms. Remarkably, they also indicated having used television programs as source of information. Only recently, the world’s largest advocacy group ‘Tourette Association of America’ (TAA) spread misinformation on GTS by promoting a reality show featuring YouTube influencer Baylen Dupree titled ‘Baylen Out Loud’, in which her symptoms were presented as tics. According to expert opinion, however, symptoms presented in numerous YouTube videos were rather similar to FTLB, although, of course, the (additional) diagnosis of tics/GTS cannot be ruled out simply based on videos^18^.

So far, no large-scale population-based study has investigated knowledge and stereotypes about GTS. More specifically, we were interested in analyzing the relationship between widespread misconceptions and media consumption for the first time. We hypothesized that the German population would have limited knowledge of GTS, including its typical symptoms and underlying causes. We presumed that Germans primarily associate GTS with coprophenomena and agree with common stereotypes. Furthermore, we speculated that stereotypical beliefs would be associated with media consumption, knowing a person with GTS, and own estimation of knowledge. Finally, we hypothesized that the spread of misinformation about GTS through the popularity of certain YouTube channels in recent years is paralleled by current stereotypical misbeliefs.

## Methods

### Data collection

**Fig. 1:**
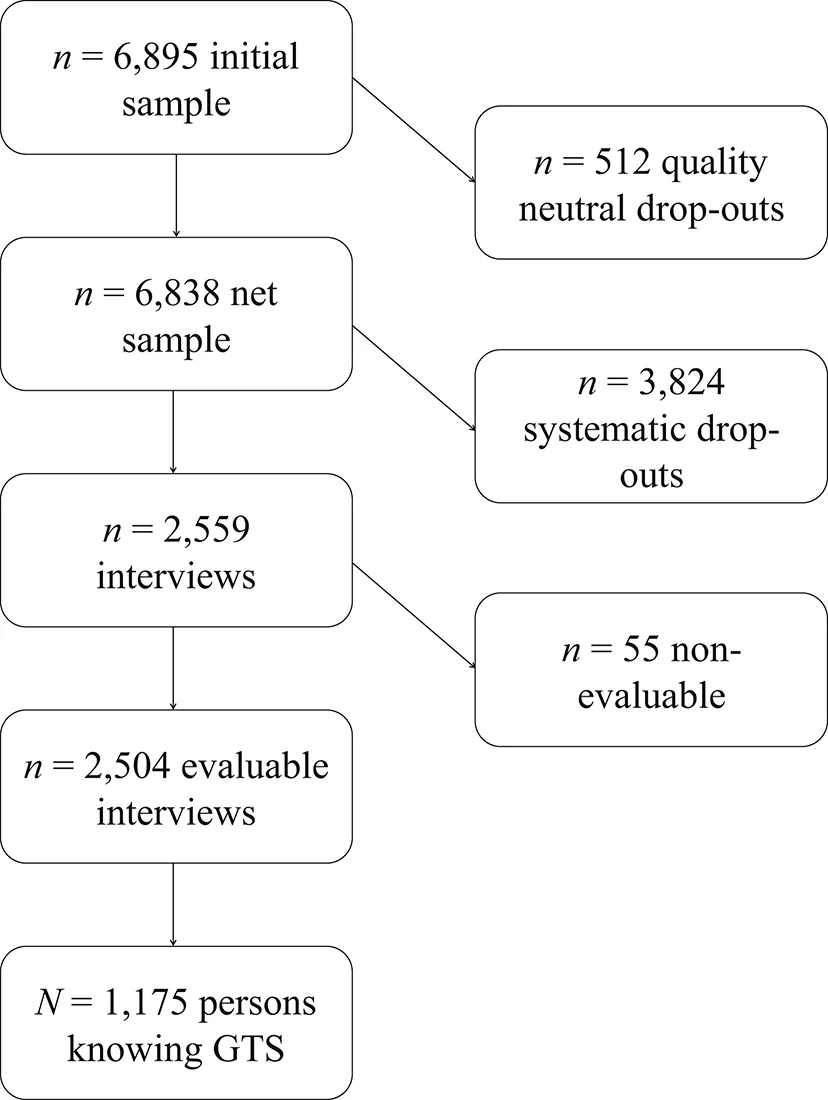
Flow-chart of in- and exclusion procedure. Note. Flow-chart illustrating initial participant selection for the interviews and the exclusion process. Quality-neutral exclusions were uninhabited apartments or selected persons not belonging to the target population, so the net sample remained. Exclusions were due to refusals, absence of the households or the selected person within, four unresponded contact attempts, holiday, sickness, or disabilities resulting in the final conducted interviews. After plausibility checks, non-evaluable participants were excluded. Out of the 2,504 evaluable participants in the final dataset, the participants knowing Gilles de la Tourette syndrome were included in our analytical sample.

For data acquisition, we collaborated with the University of Leipzig and USUMA GmbH^19^, an institution for market and social research with which we have successfully worked together before^20,21^. In USUMA’s annual nationwide survey, various institutions submit questions to the company, which collects representative data of citizens aged ≥16 years of the Federal Republic of Germany for a fee. Due to a lack of a German central register, a so-called ADM-design as the gold standard for national-wide surveys was implemented^22^. This approach guarantees that each household across the country has the same probability to be included. Based on its municipal distribution, Germany was divided into 53,000 regions consisting of 350–700 households. Out of these regions, 258 have randomly been selected and households within were chosen by random-route-procedure^23^. Within the households, an initial sample of *n* = 6,895 persons was selected with Kish-selection-grids^24^. After *n* = 512 quality neutral dropouts (*n* = 25 uninhabited apartments, *n* = 487 selected persons did not belong to the target population), a net sample of *n* = 6,383 persons remained (see Fig. 1). Out of this net sample, there were *n* = 3,824 systematic dropouts due to households refusing inquiries (*n* = 2,478), absence of the households (*n* = 593) or the selected person (*n* = 54) after four contact attempts, or the selected person being on holiday (*n* = 34), being sick or unable to participate (*n* = 37), or refusing the interview (*n* = 628). Finally, *n* = 2,559 participants filled out the questionnaires handed out by 175 trained USUMA employees from March to June 2024, of which *n* = 55 interviews were not evaluable (e.g., due to lack of formal integrity or plausibility), resulting in *n* = 2,504 evaluable interviews (Fig. 1).

### Measures

Sociodemographic data were collected face-to-face, followed by self-report questionnaires handed in by different institutions. Our survey consisted of ten questions and was developed by a panel of GTS experts from our research group. KMV developed the initial draft of the questionnaire which then was discussed and revised in a multi-step process until the panel reached an overall consensus. The German questionnaire can be found in the Supplement. To start, participants were asked, if they had ever heard or read about GTS. If not, they were excluded from the following questions. If yes, they were asked, when had heard/read about it for the first time: (i) before 2019, (ii) between 2019 and 2022, or (iii) in 2023 or later. These specific time intervals were chosen, since in 2019, a very popular German YouTube channel was launched called “Gewitter im Kopf” (in English: “thunderstorm in the brain”) with millions of subscribers, which in 2023, however, significantly lost influence^12,25^. The influencer Jan Zimmermann claimed to inform about GTS, but in fact presented FTLB^5^. Because of his enormous popularity, we speculated that this YouTube channel might had an impact on stereotypes in Germany. Accordingly, we also asked participants from which eleven sources (e.g., social media, relatives, advocacy groups) they gathered their knowledge and if they have ever seen videos of persons with presumed GTS. Furthermore, we asked to report how much time they actively spend seeking information on psychiatric diagnoses using social media.

Next, participants should estimate their overall knowledge on GTS on a five-point Likert Scale (ranging from ‘no knowledge’ to ‘very good knowledge’). In addition, participants were asked to indicate in a list with ten symptoms (see Table 1), which kind of symptoms they associate with GTS including symptoms either typical for GTS (e.g., motor and vocal tics like twitches, throat clearing, *n* = 2), or typical for FTLB (e.g., spilling drinks, violent behaviors like pushing, destroying objects, *n* = 5), or symptoms that may occur in both, GTS and FTLB (e.g., coprophenomena, *n* = 3). A list of all these symptoms can be found in Table 1. We also asked participants regarding their agreement concerning ten statements that were either true or false (see Table 2). Lastly, participants were asked to indicate whether they know a person with GTS and if yes, in which relationship they are (e.g., family member, friend).

### Scores

Based on participants’ individual responses, we calculated two scores for each participant: a knowledge score and a stereotype score, reflecting overall knowledge about GTS and the extent of misbeliefs and stereotypes, respectively.

For both scores, not answered items were removed and not taken into account. The knowledge score included twenty items (listed in Tables 1 and 2; the original German questionnaire can be found in the Supplement). It is based on the sum of correctly answered items about symptoms and general statements. Each correct answer counts one point, whereas neutral or wrong answers were given zero points. We then calculated the percentage of correctly answered items in relation to the total of answered questions.

$$\:\mathrm{K}\mathrm{n}\mathrm{o}\mathrm{w}\mathrm{l}\mathrm{e}\mathrm{d}\mathrm{g}\mathrm{e}\:\mathrm{s}\mathrm{c}\mathrm{o}\mathrm{r}\mathrm{e}=\frac{\mathrm{c}\mathrm{o}\mathrm{r}\mathrm{r}\mathrm{e}\mathrm{c}\mathrm{t}\:\mathrm{a}\mathrm{n}\mathrm{s}\mathrm{w}\mathrm{e}\mathrm{r}\mathrm{s}}{\mathrm{t}\mathrm{o}\mathrm{t}\mathrm{a}\mathrm{l}\:\mathrm{o}\mathrm{f}\:\mathrm{g}\mathrm{i}\mathrm{v}\mathrm{e}\mathrm{n}\:\mathrm{a}\mathrm{n}\mathrm{s}\mathrm{w}\mathrm{e}\mathrm{r}\mathrm{s}} \ \mathrm{*} \ 100$$

The rationale of including symptoms into the stereotype score that are either inaccurate for GTS, or can be considered undesirable or socially inappropriate was based on the sociological conceptualization of stigma^14^. Stigma is a multi-level social process^15^ in which (i) at first interpersonal differences are labeled and (ii) subsequently stereotyped by a coupling of the label with undesirable characteristics. The (ii) stereotyping can then cause (iii) the formation of groups of persons with and without the label (‘us’ and ‘them’) and (iv) status loss and discrimination of persons holding the label.

Stereotype scores were calculated by summing up concording responses to fourteen potentially stereotyping items of the categories above (e.g., funny disease, overestimation of coprophenomena; these items are all marked in Tables 1 and 2), counting one point per agreeing answer and zero points for neutral or disagreeing answers. Answers for the item ‘People with Tourette syndrome are of normal intelligence’ were inverted, meaning disagreement with this item increased the stereotype score. Once again, the percentage of the individual sum was calculated in relation to the potential maximum sum of the answered items.

$$\:\mathrm{S}\mathrm{t}\mathrm{e}\mathrm{r}\mathrm{e}\mathrm{o}\mathrm{t}\mathrm{y}\mathrm{p}\mathrm{e}\:\mathrm{s}\mathrm{c}\mathrm{o}\mathrm{r}\mathrm{e}=\frac{\mathrm{s}\mathrm{t}\mathrm{e}\mathrm{r}\mathrm{e}\mathrm{o}\mathrm{t}\mathrm{y}\mathrm{p}\mathrm{i}\mathrm{n}\mathrm{g}\:\mathrm{a}\mathrm{n}\mathrm{s}\mathrm{w}\mathrm{e}\mathrm{r}\mathrm{s}}{\mathrm{t}\mathrm{o}\mathrm{t}\mathrm{a}\mathrm{l}\:\mathrm{o}\mathrm{f}\:\mathrm{g}\mathrm{i}\mathrm{v}\mathrm{e}\mathrm{n}\:\mathrm{a}\mathrm{n}\mathrm{s}\mathrm{w}\mathrm{e}\mathrm{r}\mathrm{s}} \ \mathrm{*} \ 100$$

### Sample

The total sample consisted of 2,504 participants with an age range of 16 to 92 years (*M* = 49.68, *median* = 51, *SD* = 18.04, *n* = 1,202 males (48.00%), *n* = 1,301 females (51.96%), *n* = 1 gender diverse person (0.04%), for detailed demographics see Table S1 in the Supplement). Within the whole sample, 2.7% did not hold any scholar qualifications, 1.8% were still in school, 60.00% had a general school leave certificate (25.6% ‘Hauptschule’, 41.6% ‘mittlere Reife’/’POS’), 4.4% professional training (‘Fachschulabschluss’), 13.6% finished high school (‘Fachabitur’ and ‘Abitur’), and 10.2% held a university degree.

We filtered our sample by the question, if participants had ever heard/read of GTS, so our analytical sample consisted of *N* = 1,175 participants indicating to have heard/read of it. This sample continued with the following questions. Our analytical sample consisted of 57.4% females (*n* = 664), 42.5% males (*n* = 492), and one gender diverse person (0.09%). Age ranged from 16 to 88 years (*M* = 47.9, *median* = 48, *SD* = 16.3). Regarding education, 1.4% did not hold any qualifications, 1% was still in school, 60% had a general school leave certificate (20% ‘Hauptschule’, 40% ‘mittlere Reife’/’POS’), 4% professional training (‘Fachschulabschluss’), 17% finished high school (‘Fachabitur’ and ‘Abitur’), and 14% held a university degree.

### Statistical analyses

Analyses were performed using SPSS version 29.0.1.0. The significance threshold was set to α = 0.05. On a descriptive level, we counted who indicated to have heard/read of GTS. We report which symptoms and stereotypes participants associated or strongly associated with GTS (in %). Descriptives of the stereotype score are provided and a two-tailed Pearson correlation between knowledge and stereotype score was conducted. We used multiple linear regression models to investigate the effects of sociodemographic data (age, sex, education) and different information sources such as media types on knowledge and stereotype scores. To test for differences in knowledge and stereotype scores between persons who personally know or do not know someone with GTS, we applied two-tailed t-tests. When t-tests were significant, Cohen’s *d* was computed subsequently. Multiple linear regression models were used with the regressors sociodemographic data, confidence in own knowledge, social media consumption to get informed about psychiatric diagnoses, and whether subjects had seen a video on knowledge and stereotype scores as regressand. To test whether the time at which participants had first heard/read of GTS has impact on their knowledge or stereotypes, two separate one-way ANOVAs with both scores as dependent variable were applied. In case of significance, post hoc Tukey’ was applied.

### Ethics approval

The study was approved by the ethics committee of University of Leipzig following the Declaration of Helsinki (reference number: 008/24-ek). Prior to entering the study, participants provided written informed consent following ethical guidelines of the International Code of Marketing and Social Research Practice by the International Chamber of Commerce and the European Society for Public Opinion and Market Research.

## Results

### Who has ever heard of GTS

Out of the total sample of 2,504 participants, 46.3% (*n* = 1,175) indicated that they ever had heard of GTS, 53.7% (*n* = 1,343) never did, and four did not provide an answer. Only persons indicating knowing of GTS were included in the following analyses. Significantly more women than men indicated having heard of GTS (*Χ*^*2*^ (2) = 26.84, *p* < .001, Cramer’s *V* = 0.1). Subjects indicating having heard of GTS were significantly younger (*M* = 47.91 years, *SD* = 16.3) than those who did not (*M* = 51.2 years, *SD* = 19.28 ;*t* (2,498) = −4.55, *p* < .001, *d* = −0.18; see Figure S1 and Table S1 for age distributions in the Supplement) and had significantly higher educational attainment compared to those participants who indicated that they had never heard of GTS (*Χ* ^*2*^ (8) = 133.14, *p* < .001, Cramer’s *V* = 0.23).

### Overall knowledge and stereotypes

Table 1 shows how many of the study participants associated different symptoms with GTS, which may be typical of GTS, FTLB, or both, while Table 2 shows the agreement rates for various statements about GTS.

**Table 1:** List of all symptoms for which participants are asked to indicate the extent to which they associate them with GTS.

Item	Symptom typical for	Relative and absolute agreement rates
‘Short twitches or movements such as blinking and grimacing’	GTS	76.8% 883/1,150
‘Vocalizations like clearing one’s throat, coughing, or sniffing‘	GTS	58.7% 674/1,149
‘Spilling drinks and throwing food’*	FTLB	35.6% 409/1,149
‘Commenting on events in the surrounding area’*	FTLB	49.3% 566/1,148
‘Prolonged and/or expansive twitching or movements such as arm flailing and torso twisting‘*	both	70.7% 812/1,148
‘Destroying and throwing down objects’*	FTLB	29.2% 335/1,148
‘Pushing, hitting, or shoving other people‘*	FTLB	22.7% 261/1,148
‘Showing obscene or socially inappropriate movements or gestures’*	both	75.1% 864/1,150
‘Exclaiming curse words or insults‘*	both	87.2% 991/1,148
‘Screaming loudly‘*	both	74.9% 858/1,145

Note. GTS Gilles de la Tourette syndrome. FTLB functional tic-like behaviors. *These items were used for the calculation of the stereotype score.

**Table 2:** List of all statements regarding GTS. Participants were asked to indicate to what extent they agreed with these statements.

Item	True ✓ or False ✗	Relative frequency of agreement	Absolute frequency of agreement
‘Tourette syndrome is a neuropsychiatric disorder.’	✓	88.5%	1,014/1,146
‘Tourette syndrome is a fad or a trend.‘*	✗	2.5%	29/1,145
‘People with Tourette syndrome can pose a danger to others.’*	✗	20.7%	237/1,144
‘Many people probably have GTS, but others do not know it.’	✓	19.8%	227/1,144
‘People with Tourette syndrome are practically always noticeable everywhere because of their symptoms.‘*	✗	68.8%	788/1,146
‘Tourette syndrome is a funny disorder.’*	✗	4.2%	48/1,140
‘People with Tourette syndrome are of normal intelligence.‘*	✓	61.9%	699/1,130
‘Tourette syndrome can start at any age.’	✗	44.1%	503/1,140
‘Tourette syndrome can be caused by media consumption’*	✗	7.3%	84/1,144
‘Tourette syndrome is a chronic condition that always begins in childhood and is characterised by involuntary tics and vocalisations.‘	✓	36.4%	415/1,140

Note. GTS Gilles de la Tourette syndrome. *These items were used for the calculation of the stereotype score. The column true/false indicated how the statements were appraised for the knowledge/stereotype score.

Indicated by the knowledge score, on average, half of items were answered correctly (*n* = 1,151, *M* = 50.31%, *SD* = 12.50%, range 10–95%), while the average stereotype score was *M* = 39.78% *(n* = 1,151, *SD* = 17.08%, range 0–100%). There was a significant negative correlation between knowledge scores and stereotype scores (*r* = − .71, *p* < .001).

### Sources of information

Subjects mostly got information about GTS from television, radio, or movies (70%, *n* = 803/1,147), internet search like Google (39.3%, *n* = 449/1,143), books, newspapers or journals (35.7%, *n* = 405/1,134), followed by family members or acquaintances (30.7%, *n* = 349/1,137), social media (26.5%, *n* = 301/1,139), school or university (14.2%, *n* = 161/1,134), people with GTS (11.9%, *n* = 135), and podcasts (9.7%, *n* = 109). Only 7.8% (*n* = 88/1,132) indicated getting informed by professionals and only 1.5% (*n* = 17/1,129) by advocacy groups. Usage of other information sources was reported by 16.5% (*n* = 182/1,103).

### Knowledge and stereotypes and associated factors

The applied multiple linear regression model explaining the knowledge and using sociodemographic information source variables as regressors score did not reach significance (*F* (14, 1,053) = 1.43, *p* = .130, see Table S2 in the Supplement). In contrast, a multiple linear regression with the stereotype score as dependent variable and identical regressors reached significance (*F* (14, 1,053) = 1.73, *p* = .045, R^2^ = 0.01). Age was significantly negatively associated with stereotype scores (β = − 0.08, *t* = −2.33, *p* = .020; see Table S3 in the Supplement).

### Impact of knowing someone with GTS

Within our sample, 10.5% (*n* = 121) participants indicated to know someone with GTS personally with the following types of relationships: ‘distant friends’ (74.2%, *n* = 89), ‘close friends’ (13.3%, *n* = 16), ‘first degree relatives’ (8.3%, *n* = 10), ‘other distant relatives’ (2.5%, *n* = 3), and ‘partners’ 1.7% (*n* = 2). One person did not answer.

Knowledge scores were significantly higher in participants knowing someone with GTS (*M* = 52.11%, *SD* = 13.82%) in comparison to those who did not (*M* = 50.1% *SD* = 12.34%, *t* (1,148) = 1.68, one-sided *p* = .047, *d* = 0.162). Contrary to our assumption, there was no significant difference in stereotype scores between participants knowing someone with GTS (*M* = 42.17%, *SD* = 20.07%) in comparison to those who did not (*M* = 39.51%, *SD* = 16.69%, *t* (1,148) = 1.62, one-sided *p* = .053). However, there was a non-significant tendency towards higher stereotype scores in persons knowing someone with GTS.

### Impact of use of social media, watching videos, and estimated knowledge

To get informed about psychiatric diagnoses, participants indicated they used social media (in descending order) ‘never’ (40.4%, *n* = 462), ‘rarely’ (24.8%, *n* = 284), ‘often’ (18.8%, *n* = 215), or ‘occasionally’ (16%, *n* = 183). Thirteen participants did not answer.

Half of the sample indicated having seen a video of someone with presumed GTS (52.1%, *n* = 597), a third did not (32.9%, *n* = 377), 15.0% (*n* = 172) were not sure, and 11 did not answer (for demographics of these groups can be found in the Supplement).

Participants estimated their knowledge on GTS as follows (in descending order): ‘little knowledge’ (40.4%, *n* = 467), ‘medium knowledge’ (36.5%, *n* = 421), ‘no knowledge’ (14.1%, *n* = 163), ‘good knowledge’ (7.3%, *n* = 84), and ‘very good knowledge’ (1.7%, *n* = 20).

The regression model, which included knowledge score as the dependent variable and sociodemographic variables, estimated knowledge, having seen a video of someone with GTS, and frequency of social media use for psychiatric information as independent variables, reached statistical significance (*F* (6, 1,123) = 4.1, *p* < .001, *R*^*2*^ = 0.02, see Table 3). There was a significant positive association of age (β = 0.08, *p* = .008) and education (β = 0.09, *p* = .003) with knowledge. Frequent use of social media to get information about psychiatric diseases was positively correlated with higher knowledge percentages (β = 0.09, *p* = .004).

**Table 3:** Multiple linear regression with sociodemographic factors, estimation of own knowledge on GTS, having seen a video of someone with GTS and frequency of social media usage to get information about psychiatric diseases as independent variables, and knowledge score as dependent variable.

	B	β	t	*p*
Constant	41.96		18.59	***<0.001***
Age	0.06	0.08	2.64	***0.008***
Educational Level	0.60	0.09	2.99	***0.003***
Sex	0.16	0.01	0.22	0.828
Estimation of own knowledge	0.44	0.03	1.00	0.317
Seen video of someone with GTS	0.97	0.04	1.26	0.208
Using social media to learn about psychiatric disorders	0.97	0.09	2.85	***0.004***

Note. This regression model reached significance F (6, 1,123) = 4.1, *p* <.001, R^2^ = 0.02. Significant p-values are highlighted in bold.

The same model using the stereotype score as dependent variable instead was also significant and explained 3% of variance (*F* (6, 1,123) = 5.8, *p* < .001, *R*^*2*^ = 0.03; Table 4). In contrast to the knowledge score, age and education were significantly negatively associated with stereotype scores (age β = − 0.08, *p* = .012, education β = − 0.08, *p* = .007). Estimated knowledge was significantly positively associated with stereotype scores (β = 0.12, *p* < .001). Using social media to get informed about psychiatric diagnoses was significantly negatively associated with stereotype scores (β = − 0.08, *p* = .008). However, having seen a video of someone with GTS was significantly positively associated with stereotype scores (β = 0.06, *p* = .038).

**Table 4:** Multiple linear regression with sociodemographic factors, estimation of own knowledge on GTS, having seen a video of someone with GTS and frequency of social media usage to get information about psychiatric diseases as independent variables, and stereotype score as dependent variable.

	B	β	t	*p*
Constant	43.17		14.13	< 0.001
Age	− 0.08	− 0.08	−2.51	0.012
Educational Level	− 0.73	− 0.08	−2.69	0.007
Sex	−1.16	− 0.03	−1.14	0.256
Estimation of own knowledge	2.25	0.12	3.75	< 0.001
Seen video of someone with GTS	2.16	0.06	2.08	0.038
Using social media to learn about psychiatric disorders	−1.23	− 0.08	−2.67	0.008

Note. This multiple regression model reached significance F (6, 1,123) = 5.8, *p* <.001, R2 = 0.03. Significant p-values are highlighted in bold.

### Impact of time having first heard of GTS

47.1% (*n* = 527) of the sample indicated having heard of GTS for the first time before 2019, 11% (*n* = 123) between 2019 and 2022, and 6.3% (*n* = 71) in 2023 or later; 35.6% (*n* = 399) were unsure and 37 answers were missing.

The applied ANOVA assessing differences in knowledge scores between the groups hearing of GTS in different time periods did not reach significance (*F* (3, 1,115) = 0.61, *p* = .661, ηp^2^ **=** 0.002). Knowledge scores did not differ significantly between participants depending on time periods (‘2023 or later’ (*M* = 50.4%, *SD* = 13.77%), ‘2019 to 2022’ (*M* = 49.56%, *SD* = 12.52%), ‘before 2019’ (*M* = 50.92%, *SD* = 11.74%), ‘not sure’ (*M* = 50.02%, *SD* = 13.32%).

In contrast, the model for stereotype scores reached significance (*F* (3, 1,115) = 5.49, *p* < .001, ηp^*2*^ = 0.015): participants indicating being ‘not sure’ (their demographic details can be found in the Supplement) when having first heard of GTS (*M* = 37.10%, *SD* = 18.31%) had significantly lower stereotype scores than those who indicated having heard ‘between 2019 and 2022’ (*M =* 42.84%, *SD* = 17.53%, *p* = .006), and ‘before 2019’ (*M* = 40.56%, *SD* = 15.77%, *p* = .012), but not in comparison to those indicating having first heard of GTS ‘2023 or later’ (*M* = 42.3%, *SD* = 16.99%, *p* = .083). There were neither significant differences between subjects indicating having heard of GTS ‘before 2019’ and ‘2023 or later’ (*p* = .849), nor between ‘before 2019’ and ‘from 2019 to 2022’ (*p* = .535). Also, stereotype scores ‘between 2019 to 2022’ and ‘2023 or later’ did not differ significantly (*p* = .997).

## Discussion

This is the first large-scale population-based study investigating how well-informed Germans are about GTS and which sources of information they use. We especially focused on potential stereotypical beliefs Germans may hold and which might lead to disadvantages for individuals with GTS due to stigmatization^14,15^.

Since we investigated data from a large and representative survey, it can be concluded that only half of the German population at age 16 and above has ever heard of GTS. This is remarkable against the background that GTS is a common disorder with a prevalence rate of about 0.5%^26,27^ suggesting that nearly everyone should know someone with GTS. Thus, GTS is similar or even more common compared to other chronic disorders like schizophrenia (lifetime prevalence: 0.4%^28^) and multiple sclerosis (prevalence: 0.4%^29^), but much less known. In our opinion, this disparity can be best explained by the following factors: (i) people with GTS tend to socially isolate themselves^30^, (ii) people with GTS avoid disclosing their diagnosis to avoid stigmatization^31^, and (iii) the general public has a wrong conceptualization of GTS’ phenotype and therefore does often not recognize it^3^. This assumption is in line with recent studies^3^ and confirmed by our findings demonstrating that the majority of participants who claimed knowing GTS associated the condition with coprolalia, copropraxia, screaming, and being practically always noticeable due to associated symptoms. This, however, contrasts with data from large cohort studies in GTS demonstrating not only that coprophenomena are a rare symptom in people with GTS^32^, but also that the vast majority of people with GTS suffer from only mild to moderate tics^33^. Interestingly, some statements were correctly attributed to GTS by the majority, like stating that GTS is a neuropsychiatric developmental disorder characterized by brief twitches and movements such as blinking and grimacing. Overall, Germans have incorrect beliefs regarding their knowledge on GTS. Problematically, they also more frequently describe stereotypical ideas about GTS when they are estimating their knowledge regarding this disorder as good, which means that many may not even be aware that they have a potentially stigmatizing view of the condition.

The fact of co-occurring correct knowledge on one hand and misbeliefs on the other hand raises the questions on sources of information. Although knowing someone with GTS was associated with a statistically significantly higher level of knowledge about GTS, the effect size was so small that it is likely to be of negligible practical relevance (knowledge score of participants who know someone with GTS: M = 52.11%, SD = 13.82%; knowledge score of participants who do not know anyone with GTS: M = 50.1%, SD = 12.34%). We attribute this very weak effect primarily to the fact that there is hardly any substantial improvement in understanding through contact, as posited by Allport’s contact hypothesis^34^, since in most encounters with people with GTS, people do simply not recognize that the other person has GTS.

Although we found that the use of social media to obtain information about psychiatric disorders was statistically significantly associated with greater knowledge and fewer stereotypes, the observed effects were very small (|β| < 0.10), and their practical relevance should therefore be interpreted with caution. However, having seen a video depicting an individual purported to have GTS was associated with greater endorsement of stereotypes but was not associated with improved knowledge. It therefore can be speculated that in particular video portrays of people with supposed GTS often are erroneously depicting FTLB phenomenology instead of GTS symptoms^11^. In line with this assumption, it has been demonstrated that there is a generally higher public interest in YouTube videos showing more severely affected and extreme cases of presumed GTS^10^. The observed effect was very small, suggesting that, based on our data, changes to the (mis)representations of GTS patients on social media are unlikely to substantially reduce stereotypical perceptions among Germans. Therefore, interventions targeting social media portrayals of GTS patients are unlikely to result in meaningful improvements in current public perceptions of GTS. Rizzo et al.^35^ discussed risks and benefits of rising social media content regarding psychiatric diagnoses as a ‘two-sided sword’ offering possibilities for exchange and raising awareness, but also harmful potential by perpetuating stereotypes. However, it is important to mention that also persons with FTLB probably suffer from consequences of stereotyping about GTS when being labeled as someone with GTS in social media or when being misdiagnosed^18^.

Remarkably, a coexistence of correct knowledge on one hand and stigmatization on the other hand has also been observed in healthcare professionals even though personal contact points exist. According to a cross-sectional online survey in the UK including 199 participants with GTS, participants reported to feel discriminated by not being taken seriously at consulted professionals^36^ indicating that personal contact and knowledge alone do not necessarily prevent from stigmatization.

Contrary to our hypothesis, we found no relevant association between the time when participants first heard of GTS and their level of knowledge about GTS. However, stereotype scores were significant lower when Germans were unsure when they first had heard of GTS in comparison to both having first heard of GTS between 2019 and 2022 and before 2019. This pattern may indicate that participants with minimal or vague prior exposure have not internalized stereotypical portrayals, whereas those with earlier exposure — possibly through media or social media — may have encountered more stereotyped or sensationalized representations. We speculate that the release of the German YouTube channel “Gewitter im Kopf” who was most popular between 2019 and 2022 — as well as similar further TikTok and YouTube videos — providing mostly incorrect and misleading information might have further fueled misbeliefs and stereotypes of GTS. At the same time, it is important to emphasize at this point that the operationalization of the potential influence of the YouTube channels like ‘Gewitter im Kopf’ – by asking when respondents first heard of GTS – merely represents a probabilistic association that is influenced by other third-party variables, which makes interpretation more difficult.

Overall, our data confirm severe misconceptions of GTS in Germans. This can lead to a broad variety of unfair disadvantages for people with tics. Interpersonally, these stereotypes may cause ‘othering’^15^ and social isolation. On a personal level, it may result in internalized self-stigma with individuals trying to hide one’s symptoms and deciding not to talk about the diagnosis, potentially entailing further self-initiated isolation. Hiding diagnoses prevents affected persons from receiving needed support and compensation e.g., in school or at work^37^. Thus, stereotypical beliefs may hinder societal participation and lead to inequality on several levels, e.g., employment, healthcare, and relationships^36^. Since attention deficit/hyperactivity disorder, obsessive–compulsive disorder, depression, anxiety, functional neurological disorder, and autism are well-known and common comorbidities in GTS^32,38,39^, it can be speculated that societal stigmatization of people with GTS may contribute to the development and maintenance of comorbid psychopathology due to loneliness^40^, smaller social network size^41^, unemployment^42,43^, and poverty^44^. Comorbidities and tic severity have been shown to be closely interwoven and to have substantial influence on quality of life^45,46^.

Structural stigmatization cannot be solved on the individual level. Instead, public societal images of GTS must be addressed^14^. The results of our study clearly indicate the need for information campaigns, for example in educational institutions but also for peers^47^, in order to reduce prejudices among the German population. Research has already started to develop educational interventions, however, not all of them were capable of reducing stereotypical thinking and social exclusion^48–53^. Future studies should use experimental designs to identify which factors lead to stigmatizations in order to tailor interventions for stigma reduction^52^.

The major strength of this study is the large sample, which was based on a highly representative survey that was conducted at great expense by visiting respondents throughout Germany. Limitations of the study are: (i) the questionnaire used was not validated. However, it was created by our research group of GTS experts in a multi-step process; (ii) due to associated relevant costs, the number of questions was restricted to a minimum; (iii) we examined only attitudes towards GTS, but not whether respondents actively discriminate against persons with GTS on a behavioral level; (iv) our study design does not allow for causal conclusions, however, future studies could utilize additional methods, such as graphical causal models, to gain further insights from the data^54,55^, (v) we included only people *≥* 16 years and therefore no statement can be made about knowledge and stereotypes and the influence of social media in younger people, (vi) the associations between GTS knowledge and stereotypical beliefs about GTS on the one side and other variables on the other were relatively small, and (vii) even though the ADM design for participant selection was conducted to gain a representative overall sample, the results might be prone to non-response bias.

## Supplementary Information

Below is the link to the electronic supplementary material.


Supplementary Material 1



Supplementary Material 2


## Data Availability

The datasets generated during and/or analyzed during the current study are available from the corresponding author on reasonable request.
