# Supplementary material for: Stereotypes about tourette syndrome and associations with social media use: results from a large-scale german population survey
Source: Sci Rep. 2026 Aug 28;16:27409. doi: 10.1038/s41598-026-66366-8 (PMC13534477; doi:10.1038/s41598-026-66366-8)
Supplement: Supplementary file 1 [file 41598_2026_66366_MOESM1_ESM.pdf]

Supplement: Stereotypes about Gilles de la Tourette Syndrome and associations with media use: Results from a Large-Scale Representative German Population Study

C Cramer †[1], S Schmitt †*[1,2], N Szejko[1,3], K R Müller-Vahl[1]

†Contributed equally

*Corresponding author

[1]Clinic of Psychiatry, Social Psychiatry and Psychotherapy, Hannover Medical School, Hannover, Germany

[2]Department of Psychiatry, Psychotherapy and Psychosomatics, RWTH Aachen University, Aachen, Germany

[3]Institute for Translational Neuroscience and Clinical Psychology, RWTH Aachen, Aachen, Germany

[4]Department of Health Sociology, Education and Medical Communication, Institute of Mother and Child, Warsaw, Poland

Table S1

Sociodemographics

	**Overall sample**	**Analytical Sample**
**N (%)**	2,504 (100)	1,175 (46.3)
**Age**		
range	16-92	16-88
M (SD)	49.68 (18.04)	47.91 (16.3)
**Distribution n (%)**		
16-24	250 (9.98)	104 (8.99)
25-34	366 (14.62)	171 (14.78)
35-44	417 (16.65)	227 (19.62)
45-54	379 (15.14)	209 (18.06)
55-64	478 (19.09)	250 (21.61)
65-74	409 (16.33)	147 (12.71)
≥ 75	205 (8.19)	49 (4.24)
**Sex**		
Female	1,301 (51.96)	664 (57.4)
Male	1,202 (48)	492 (42.5)
Diverse	1 (0.04)	1 (0.09)
**Education**		
In School	48 (1.93)	17 (1.48)
Haupt/Volksschulabschluss	637 (25.56)	235 (20.4)
Mittlere Reife/Realschule	847 (33.99)	414 (35.94)
Polytechnische Oberschule (POS)	189 (7.58)	48 (4.17)
Fachschulabschluss	109 (4.37)	55 (4.77)
High school leave	339 (13.60)	205 (17.8)
University degree	255 (10.23)	162 (14.06)
Other certificates	1 (0.04)	0
No degree	67 (2.69)	16 (1.39)
**Nationality**		
German	2.387 (95.86)	1,137 (98.61)
Not German	103 (4.14)	16 (1.39)
**Employment**		
Full-time (>35h)	1,099 (44.24)	550 (48.08)

Part-time (15-24h)	296 (11.92)	169 (14.77)
Hour-wise	73 (2.94)	53 (4.63)
unemployed	15 (0.6)	43 (3.76)
Retired	650 (26.17)	214 (18.71)
Stay-at-home	63 (2.54)	35 (3.06)
Vocational training	47 (1.89)	19 (1.66)
In school	127 (5.11)	61 (5.33)
**Profession**		
None	165 (6.66)	65 (5.7)
Worker	284 (11.46)	80 (7.01)
Skilled worker	266 (10.73)	70 (6.13)
Independent farmer	9 (0.36)	1 (0.09)
Liberal profession	37 (1.49)	18 (1.58)
Self-employed	123 (4.96)	61 (5.36)
Employed	1,471 (59.34)	766 (67.13)
Civil servant	124 (5)	80 (7.01)
**Marital status**		
single	788 (31.47)	391 (33.79)
Married / living together	1,107 (44.21)	519 (44.86)
Married / living separatly	40 (1.6)	17 (1.47)
Divorced	332 (13.26)	161 (13.92)
Widowed	237 (9.46)	69 (5.96)
**Children <16 years**		
0	2,014 (80.75)	919 (79.71)
1	293 (11.75)	133 (11.54)
2	157 (6.3)	84 (7.29)
3	25 (1)	15 (1.3)
4	5 (0.2)	2 (0.17)
**Income (Household €/month)**		
< 750	32 (1.3)	11 (0.97)
750 - 1,250	180 (7.28)	72 (6.34)
1,250 - < 2,000	446 (18.05)	180 (15.86)
≥ 2,000	1,813 (73.37)	872 (76.83)
**Religion**		
Protestant	885 (35.46)	455 (39.39)
Catholic	687 (27.52)	357 (30.91)
Muslim	69 (2.76)	12 (1.04)
Other	51 (2.04)	16 (1.39)
None	804 (32.21)	315 (27.27)
**Location**		
Western Germany	2,002 (79.98)	1,026 (88.68)
Eastern Germany	501 (20.02)	131 (11.32)

Table S2

Multiple linear regression with information sources and sociodemographics (age, sex, educational level) as independent variables and knowledge score as dependent variable did not reach significance.

	B	β	*t*	*p*
Constant	47.39		16.01	<.001
Age	0.04	.05	1.53	.127
Educational Level	0.55	.08	2.59	**.010**
Sex	0.02	.00	0.02	.982
Social Media	0.32	.03	0.69	.493
Internet	-0.25	-.02	-0.599	.550
Advocacy groups	-3.97	-.11	-3.07	**.002**
Family and friends	0.13	.01	0.35	.724
TV, radio, movies	0.20	.02	0.54	.592
Podcasts	0.37	.02	0.60	.548
Books, newspaper, journals	0.26	.02	0.66	.509
People with GTS	0.03	.00	0.06	.954
School, university	0.28	.02	0.51	.613
Professionals	0.59	.03	0.86	.388
Other	-0.15	-.01	-0.34	.731

Note. This multiple regression model did not reach significance (F (14, 1,053) = 1.43, p = .130). Significant p-values are marked in bold letters.

Table S3

Multiple linear regression with information sources as independent variables and stereotype score as dependent variable was significant.

	B	β	*t*	*p*
Constant	35.21		13.64	<.001
Age	-0.09	-.08	-2.33	**.020**
Educational Level	-0.36	-.04	-1.26	.208
Sex	-1.04	-.03	-0.97	.331
Social Media	0.05	.00	0.07	.942
Internet	0.59	.04	1.03	.303
Advocacy groups	0.37	.01	0.22	.829
Family and friends	-0.81	-.05	-1.58	.114
TV, radio, movies	0.63	.04	1.25	.212
Podcasts	-1.46	-.06	-1.73	.085
Books, newspaper, journals	0.98	.06	1.85	.064
People with GTS	1.00	.05	1.43	.153
School, university	-0.89	-.05	-1.19	.234
Professionals	1.47	.06	1.57	.117
Other	0.45	.02	0.69	.491

Note. This multiple regression model reached significance F (14, 1,053) = 1.73, $p = .045$, $R^2 = .01$. Significant p-values are highlighted in bold.

Supplementary Table S4. Demographics of participants grouped by when they have first heard of GTS.

N (%)	2023 or later<br>71 ()	2019-2022<br>123 ()	Before 2019<br>525 ()	Not sure<br>398 ()
**Age**				
range	16-82	16-88	16-84	16-88
M (SD)	40.53 (18.71)	40.15 (16.90)	49.87 (15.44)	49.36 (15.74)
**Sex N (%)**				
Female	40 (56.34)	67 (54.5)	303 (57.7)	232 (58.1)
Male	30 (42.25)	56 (45.5)	224 (42.5)	167 (41.9)
Divers	1 (1.41)	0	0	0
**Education**				
In School	7 (10.0)	1 (0.8)	3 (0.6)	6 (1.5)
Haupt/Volksschulabschluss	11 (15.7)	24 (19.5)	92 (17.5)	103 (25.9)
Mittlere Reife/Realschule	24 (34.3)	50 (40.7)	185 (35.2)	141 (35.4)
Polytechnische Oberschule	5 (7.1)	1 (0.8)	20 (3.8)	22 (5.5)
Fachschulabschluss	3 (4.3)	8 (6.5)	33 (6.3)	10 (2.5)
High school leave	16 (22.9)	23 (18.7)	96 (18.3)	61 (15.3)
University degree	4 (5.7)	16 (13.0)	92 (17.5)	43 (10.8)
No degree	0	0	4 (0.8)	12 (3.0)

Note. Demographics included means and standard deviations of age, absolute numbers and percentages of sex, and absolute numbers and percentages of educational levels separately of participants depending on when they first heard of GTS.

Supplementary Table S5. Demographics of participants who have seen a video of someone with supposed GTS, participants who did not, and participants who were not sure about it.

N (%)	Yes<br>597 (52.1)	No<br>377 (32.9)	Not sure<br>172 (15.0)
Age			
range	16-86	18-88	16-86
M (SD)	46.13 (16.59)	49.43 (15.87)	50.03 (15.49)
Sex N (%)			
Female	356 (59.63)	208 (55.17)	96 (55.81)
Male	240 (40.20)	169 (44.83)	76 (44.19)
Divers	1 (0.17)	0	0

**Education**			
**In School**	**9 (1.52)**	**4 (1.07)**	**4 (2.33)**
**Haupt/Volksschulabschluss**	**117 (19.70)**	**80 (21.33)**	**34 (19.77)**
**Mittlere Reife/Realschule**	**198 (33.33)**	**144 (38.40)**	**68 (39.53)**
**Polytechnische Oberschule**	**20 (3.37)**	**18 (4.80)**	**10 (5.81)**
**Fachschulabschluss**	**28 (4.71)**	**21 (5.60)**	**6 (3.49)**
**High school leave**	**119 (20.03)**	**56 (14.93)**	**29 (16.86)**
**University degree**	**96 (16.16)**	**44 (11.73)**	**20 (11.63)**
**No degree**	**7 (1.18)**	**8 (2.13)**	**1 (0.58)**

Note. Demographics included means and standard deviations of age, absolute numbers an percentages of sex, and absolute numbers and percentages of educational levels separately of participants who responded 'yes', 'no', and 'not sure' to the item: 'Did you ever watch a movie, a documentary or a video with a person with supposed GTS?'.

Figure S1

Violin plots of age distributions of participants who have heard of GTS and those who did not

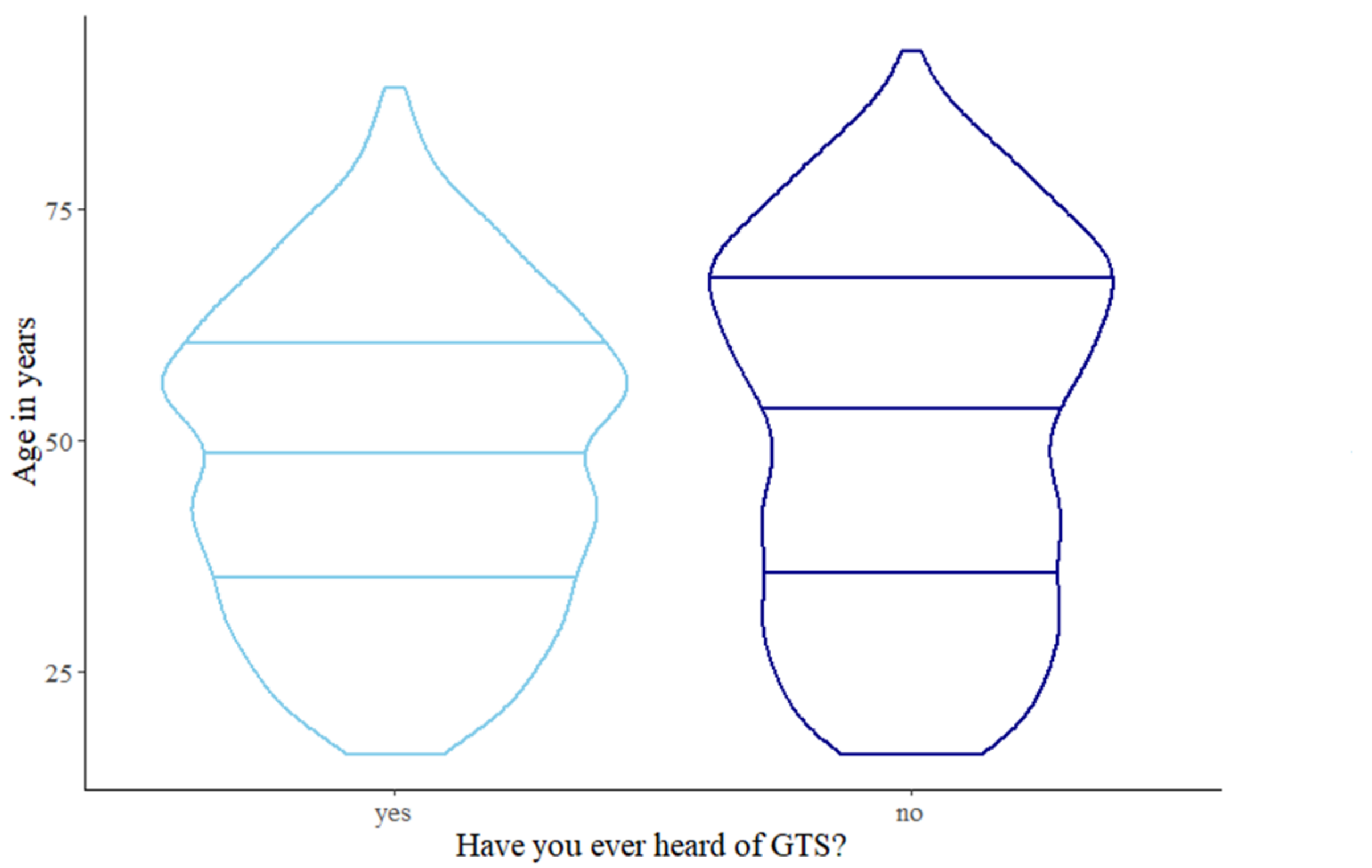

Violin plots showing the age distribution (y-axis, in years) of participants who had heard of GTS (light blue) and those who had not heard of GTS (dark blue). Horizontal lines within the violin plots indicate the 25th, 50th (median), and 75th percentiles.

J01.	Haben Sie jemals vom „Tourette-Syndrom“ gehört oder gelesen?	ja .... ☐<br>nein .... ☐	 ➔ weiter mit **Frage K01**

J02.	Wann haben Sie zum ersten Mal vom Tourette-Syndrom gehört?	im Jahr 2023 oder danach .... ☐<br>zwischen 2019 und 2022 .... ☐<br>vor 2019 .... ☐<br>Ich bin mir nicht sicher .... ☐

Aus welchen der folgenden Quellen haben Sie Ihr Wissen zum Tourette-Syndrom?

J03.	*Bitte machen Sie in <u>jeder Zeile</u> NUR EIN KREUZ.*	trifft überhaupt nicht zu	trifft eher nicht zu	trifft eher zu	trifft voll und ganz zu
01	Soziale Medien wie Facebook, Instagram, X (ehem. Twitter), Pinterest, YouTube, Snapchat, TikTok, LinkedIn, Xing	☐	☐	☐	☐
02	Internet, z.B. Google	☐	☐	☐	☐
03	Selbsthilfegruppen wie TGD e.V., IVTS e.V., LifeTiccer e.V.	☐	☐	☐	☐
04	Familie und Bekannte	☐	☐	☐	☐
05	Berichte in TV, Radio und Kino	☐	☐	☐	☐
06	Podcasts	☐	☐	☐	☐
07	Bücher, Zeitungen und Zeitschriften	☐	☐	☐	☐
08	Von Betroffenen mit Tourette-Syndrom	☐	☐	☐	☐
09	Schule, Studium	☐	☐	☐	☐
10	Fachpersonal, etwa Ärzte/innen und Psycholog/innen	☐	☐	☐	☐
11	sonstiges	☐	☐	☐	☐

J04.	kein Wissen<br>1	2	3	4	sehr gutes Wissen<br>5
Wie schätzen Sie Ihr Wissen zum Tourette-Syndrom ein?	☐	☐	☐	☐	☐

51

Welches der nachfolgend genannten Merkmale verbinden Sie wie stark mit dem Tourette-Syndrom?

J05.	*Bitte machen Sie in jeder Zeile NUR EIN KREUZ.*	verbinde ich sehr stark …	verbinde ich stark …	verbinde ich eher schwach …	verbinde ich gar nicht …
		… mit dem Tourette-Syndrom			
01	Kurze Zuckungen bzw. Bewegungen wie Augen blinzeln und Grimassieren	☐	☐	☐	☐
02	Auskippen von Getränken und Werfen von Lebensmitteln	☐	☐	☐	☐
03	Zeigen obszöner oder sozial unpassender Bewegungen oder Gesten	☐	☐	☐	☐
04	Kommentieren von Geschehnissen in der Umgebung	☐	☐	☐	☐
05	Lautäußerungen wie Räuspern, Husten, Schniefen	☐	☐	☐	☐
06	Ausrufen von Schimpfwörtern und Beleidigungen	☐	☐	☐	☐
07	Länger dauernde und/oder ausladende Zuckungen bzw. Bewegungen wie Arm schleudern und Rumpfverdrehen	☐	☐	☐	☐
08	Laute Schreie	☐	☐	☐	☐
09	Zerstören und Herunterwerfen von Gegenständen	☐	☐	☐	☐
10	Stoßen, Schlagen und Schubsen anderer Personen	☐	☐	☐	☐

J06.	Haben Sie jemals einen Film, eine Dokumentation oder ein Video gesehen, in dem eine Person auftrat, die das Tourette-Syndrom hatte?	ja.. ☐<br>nein.. ☐<br>weiß nicht .... ☐

J07.	Kennen Sie persönlich eine Person mit Tourette-Syndrom?	ja.. ☐<br>nein.................................... ☐ ➔ weiter mit **Frage J09**

J08.	In welcher Beziehung stehen Sie zu der Person mit Tourette-Syndrom, die Sie persönlich kennen?	Ich selbst habe das Tourette-Syndrom. ..... ☐<br>Angehörige ersten Grades: Eigenes Kind, Geschwister, Eltern .... ☐<br>weitere Verwandtschaft .... ☐<br>Partner/in .... ☐<br>gute/r Freund/in .... ☐<br>entferntere Bekannte .... ☐

52

J09.	*Inwiefern stimmen Sie den folgenden Aussagen zu?*	stimme völlig zu	stimme zu	stimme weder zu noch nicht zu	stimme nicht zu	stimme über-haupt nicht zu
01	Das Tourette-Syndrom ist eine neurologisch-psychiatrische Erkrankung.	☐	☐	☐	☐	☐
02	Das Tourette-Syndrom ist eine Modeerscheinung bzw. ein Trend.	☐	☐	☐	☐	☐
03	Menschen mit Tourette-Syndrom können für Andere eine Gefahr darstellen.	☐	☐	☐	☐	☐
04	Vermutlich haben viele Menschen Tourette-Syndrom, von denen man es gar nicht weiß.	☐	☐	☐	☐	☐
05	Menschen mit Tourette-Syndrom fallen wegen ihrer Symptome praktisch immer und überall auf.	☐	☐	☐	☐	☐
06	Tourette-Syndrom ist eine lustige Erkrankung.	☐	☐	☐	☐	☐
07	Menschen mit Tourette-Syndrom sind normal intelligent.	☐	☐	☐	☐	☐
08	Das Tourette-Syndrom kann in jedem Lebensalter auftreten.	☐	☐	☐	☐	☐
09	Das Tourette-Syndrom kann durch Medienkonsum hervorgerufen werden.	☐	☐	☐	☐	☐
10	Das Tourette-Syndrom ist eine chronisch verlaufende Erkrankung, die stets in der Kindheit beginnt und durch unwillkürliche Zuckungen und Lautäußerungen gekennzeichnet ist.	☐	☐	☐	☐	☐

J10.	Wie häufig nutzen Sie soziale Medien (z.B. Instagram, YouTube, TikTok, Facebook, Twitch), um sich aktiv über psychiatrische Erkrankungen zu informieren?	nutze ich häufig	☐
		nutze ich gelegentlich	☐
		nutze ich nur selten	☐
		nutze ich nie	☐

53
