# Supplementary material for: Stereotypes about tourette syndrome and associations with social media use: results from a large-scale german population survey
Source: Sci Rep. 2026 Aug 28;16:27409. doi: 10.1038/s41598-026-66366-8 (PMC13534477; doi:10.1038/s41598-026-66366-8)
Supplement: Supplementary file 2 [file 41598_2026_66366_MOESM2_ESM.docx]

**Supplement: Stereotypes about Gilles de la Tourette Syndrome and associations with media use: Results from a Large-Scale Representative German Population Study**

C Cramer †^1^, S Schmitt †*^1,2^, N Szejko^1,3^, K R Müller-Vahl^1^

†Contributed equally

*Corresponding author

^1^Clinic of Psychiatry, Social Psychiatry and Psychotherapy, Hannover Medical School, Hannover, Germany

^2^Department of Psychiatry, Psychotherapy and Psychosomatics, RWTH Aachen University, Aachen, Germany

^3^Institute for Translational Neuroscience and Clinical Psychology, RWTH Aachen, Aachen, Germany

^4^Department of Health Sociology, Education and Medical Communication, Institute of Mother and Child, Warsaw, Poland

**Table S1**

*Sociodemographics*

|  | **Overall sample** | **Analytical Sample** |
| --- | --- | --- |
| **N (%)** | 2,504 (100) | 1,175 (46.3) |
| **Age** |  |  |
| range | 16-92 | 16-88 |
| M (SD) | 49.68 (18.04) | 47.91 (16.3) |
| **Distribution n (%)** |  |  |
| 16-24 | 250 (9.98) | 104 (8.99) |
| 25-34 | 366 (14.62) | 171 (14.78) |
| 35-44 | 417 (16.65) | 227 (19.62) |
| 45-54 | 379 (15.14) | 209 (18.06) |
| 55-64 | 478 (19.09) | 250 (21.61) |
| 65-74 | 409 (16.33) | 147 (12.71) |
| ≥ 75 | 205 (8.19) | 49 (4.24) |
| **Sex** |  |  |
| Female | 1,301 (51.96) | 664 (57.4) |
| Male | 1,202 (48) | 492 (42.5) |
| Diverse | 1 (0.04) | 1 (0.09) |
| **Education** |  |  |
| In School | 48 (1.93) | 17 (1.48) |
| Haupt/Volksschulabschluss | 637 (25.56) | 235 (20.4) |
| Mittlere Reife/Realschule | 847 (33.99) | 414 (35.94) |
| Polytechnische Oberschule (POS) | 189 (7.58) | 48 (4.17) |
| Fachschulabschluss | 109 (4.37) | 55 (4.77) |
| High school leave | 339 (13.60) | 205 (17.8) |
| University degree | 255 (10.23) | 162 (14.06) |
| Other certificates | 1 (0.04) | 0 |
| No degree | 67 (2.69) | 16 (1.39) |
| **Nationality** |  |  |
| German | 2.387 (95.86) | 1,137 (98.61) |
| Not German | 103 (4.14) | 16 (1.39) |
| **Employment** |  |  |
| Full-time (>35h) | 1,099 (44.24) | 550 (48.08) |
| Part-time (15-24h) | 296 (11.92) | 169 (14.77) |
| Hour-wise | 73 (2.94) | 53 (4.63) |
| unemployed | 15 (0.6) | 43 (3.76) |
| Retired | 650 (26.17) | 214 (18.71) |
| Stay-at-home | 63 (2.54) | 35 (3.06) |
| Vocational training | 47 (1.89) | 19 (1.66) |
| In school | 127 (5.11) | 61 (5.33) |
| **Profession** |  |  |
| None | 165 (6.66) | 65 (5.7) |
| Worker | 284 (11.46) | 80 (7.01) |
| Skilled worker | 266 (10.73) | 70 (6.13) |
| Independent farmer | 9 (0.36) | 1 (0.09) |
| Liberal profession | 37 (1.49) | 18 (1.58) |
| Self-employed | 123 (4.96) | 61 (5.36) |
| Employed | 1,471 (59.34) | 766 (67.13) |
| Civil servant | 124 (5) | 80 (7.01) |
| **Marital status** |  |  |
| single | 788 (31.47) | 391 (33.79) |
| Married / living together | 1,107 (44.21) | 519 (44.86) |
| Married / living separatly | 40 (1.6) | 17 (1.47) |
| Divorced | 332 (13.26) | 161 (13.92) |
| Widowed | 237 (9.46) | 69 (5.96) |
| **Children <16 years** |  |  |
| 0 | 2,014 (80.75) | 919 (79.71) |
| 1 | 293 (11.75) | 133 (11.54) |
| 2 | 157 (6.3) | 84 (7.29) |
| 3 | 25 (1) | 15 (1.3) |
| 4 | 5 (0.2) | 2 (0.17) |
| **Income (Household €/month)** | |  |
| < 750 | 32 (1.3) | 11 (0.97) |
| 750 - 1,250 | 180 (7.28) | 72 (6.34) |
| 1,250 - < 2,000 | 446 (18.05) | 180 (15.86) |
| ≥ 2,000 | 1,813 (73.37) | 872 (76.83) |
| **Religion** |  |  |
| Protestant | 885 (35.46) | 455 (39.39) |
| Catholic | 687 (27.52) | 357 (30.91) |
| Muslim | 69 (2.76) | 12 (1.04) |
| Other | 51 (2.04) | 16 (1.39) |
| None | 804 (32.21) | 315 (27.27) |
| **Location** |  |  |
| Western Germany | 2,002 (79.98) | 1,026 (88.68) |
| Eastern Germany | 501 (20.02) | 131 (11.32) |

| **Table S2**  *Multiple linear regression with information sources and sociodemographics (age, sex, educational level) as independent variables and knowledge score as dependent variable did not reach significance.* | | | | |
| --- | --- | --- | --- | --- |
|  | B | β | *t* | *p* |
| Constant | 47.39 |  | 16.01 | <.001 |
| Age | 0.04 | .05 | 1.53 | .127 |
| Educational Level | 0.55 | .08 | 2.59 | **.010** |
| Sex | 0.02 | .00 | 0.02 | .982 |
| Social Media | 0.32 | .03 | 0.69 | .493 |
| Internet | -0.25 | -.02 | -0.599 | .550 |
| Advocacy groups | -3.97 | -.11 | -3.07 | **.002** |
| Family and friends | 0.13 | .01 | 0.35 | .724 |
| TV, radio, movies | 0.20 | .02 | 0.54 | .592 |
| Podcasts | 0.37 | .02 | 0.60 | .548 |
| Books, newspaper, journals | 0.26 | .02 | 0.66 | .509 |
| People with GTS | 0.03 | .00 | 0.06 | .954 |
| School, university | 0.28 | .02 | 0.51 | .613 |
| Professionals | 0.59 | .03 | 0.86 | .388 |
| Other | -0.15 | -.01 | -0.34 | .731 |

*Note. This multiple regression model did not reach significance (F (14, 1,053) = 1.43, p = .130). Significant p-values are marked in bold letters.*

| **Table S3**  *Multiple linear regression with information sources as independent variables and stereotype score as dependent variable was significant.* | | | | |
| --- | --- | --- | --- | --- |
|  | B | β | *t* | *p* |
| Constant | 35.21 |  | 13.64 | <.001 |
| Age | -0.09 | -.08 | -2.33 | **.020** |
| Educational Level | -0.36 | -.04 | -1.26 | .208 |
| Sex | -1.04 | -.03 | -0.97 | .331 |
| Social Media | 0.05 | .00 | 0.07 | .942 |
| Internet | 0.59 | .04 | 1.03 | .303 |
| Advocacy groups | 0.37 | .01 | 0.22 | .829 |
| Family and friends | -0.81 | -.05 | -1.58 | .114 |
| TV, radio, movies | 0.63 | .04 | 1.25 | .212 |
| Podcasts | -1.46 | -.06 | -1.73 | .085 |
| Books, newspaper, journals | 0.98 | .06 | 1.85 | .064 |
| People with GTS | 1.00 | .05 | 1.43 | .153 |
| School, university | -0.89 | -.05 | -1.19 | .234 |
| Professionals | 1.47 | .06 | 1.57 | .117 |
| Other | 0.45 | .02 | 0.69 | .491 |

*Note. This multiple regression model reached significance F (14, 1,053) = 1.73, p = .045, R^2^ = .01. Significant p-values are highlighted in bold.*

**Supplementary Table S4.** Demographics of participants grouped by when they have first heard of GTS.

|  | **2023 or later** | **2019-2022** | **Before 2019** | **Not sure** |
| --- | --- | --- | --- | --- |
| **N (%)** | 71 () | 123 () | 525 () | 398 () |
| **Age** |  |  |  |  |
| range | 16-82 | 16-88 | 16-84 | 16-88 |
| M (SD) | 40.53 (18.71) | 40.15 (16.90) | 49.87 (15.44) | 49.36 (15.74) |
| **Sex N (%)** |  |  |  |  |
| Female | 40 (56.34) | 67 (54.5) | 303 (57.7) | 232 (58.1) |
| Male | 30 (42.25) | 56 (45.5) | 224 (42.5) | 167 (41.9) |
| Divers | 1 (1.41) | 0 | 0 | 0 |
| **Education** |  |  |  |  |
| In School | 7 (10.0) | 1 (0.8) | 3 (0.6) | 6 (1.5) |
| Haupt/Volksschulabschluss | 11 (15.7) | 24 (19.5) | 92 (17.5) | 103 (25.9) |
| Mittlere Reife/Realschule | 24 (34.3) | 50 (40.7) | 185 (35.2) | 141 (35.4) |
| Polytechnische Oberschule | 5 (7.1) | 1 (0.8) | 20 (3.8) | 22 (5.5) |
| Fachschulabschluss | 3 (4.3) | 8 (6.5) | 33 (6.3) | 10 (2.5) |
| High school leave | 16 (22.9) | 23 (18.7) | 96 (18.3) | 61 (15.3) |
| University degree | 4 (5.7) | 16 (13.0) | 92 (17.5) | 43 (10.8) |
| No degree | 0 | 0 | 4 (0.8) | 12 (3.0) |

*Note. Demographics included means and standard deviations of age, absolute numbers and percentages of sex, and absolute numbers and percentages of educational levels separately of participants depending on when they first heard of GTS.*

**Supplementary Table S5. Demographics of participants who have seen a video of someone with supposed GTS, participants who did not, and participants who were not sure about it.**

|  | **Yes** | **No** | **Not sure** |
| --- | --- | --- | --- |
| **N (%)** | **597 (52.1)** | **377 (32.9)** | **172 (15.0)** |
| **Age** |  |  |  |
| **range** | **16-86** | **18-88** | **16-86** |
| **M (SD)** | **46.13 (16.59)** | **49.43 (15.87)** | **50.03 (15.49)** |
| **Sex N (%)** |  |  |  |
| **Female** | **356 (59.63)** | **208 (55.17)** | **96 (55.81)** |
| **Male** | **240 (40.20)** | **169 (44.83)** | **76 (44.19)** |
| **Divers** | **1 (0.17)** | **0** | **0** |
| **Education** |  |  |  |
| **In School** | **9 (1.52)** | **4 (1.07)** | **4 (2.33)** |
| **Haupt/Volksschulabschluss** | **117 (19.70)** | **80 (21.33)** | **34 (19.77)** |
| **Mittlere Reife/Realschule** | **198 (33.33)** | **144 (38.40)** | **68 (39.53)** |
| **Polytechnische Oberschule** | **20 (3.37)** | **18 (4.80)** | **10 (5.81)** |
| **Fachschulabschluss** | **28 (4.71)** | **21 (5.60)** | **6 (3.49)** |
| **High school leave** | **119 (20.03)** | **56 (14.93)** | **29 (16.86)** |
| **University degree** | **96 (16.16)** | **44 (11.73)** | **20 (11.63)** |
| **No degree** | **7 (1.18)** | **8 (2.13)** | **1 (0.58)** |

***Note. Demographics included means and standard deviations of age, absolute numbers an percentages of sex, and absolute numbers and percentages of educational levels separately of participants who responded ‘yes’, ‘no’, and ‘not sure’ to the item: ‘Did you ever watch a movie, a documentary or a video with a person with supposed GTS?’.***

**Figure S1**

*Violin plots of age distributions of participants who have heard of GTS and those who did not*

Violin plots showing the age distribution (y-axis, in years) of participants who had heard of GTS (light blue) and those who had not heard of GTS (dark blue). Horizontal lines within the violin plots indicate the 25^th^, 50^th^ (median), and 75^th^ percentiles.

The German questionnaire used
